# Physical activity and content in a variety of physically active learning: an observational case study of real-world practices

**DOI:** 10.3389/fspor.2024.1504704

**Published:** 2025-01-03

**Authors:** Jan-Michael Johansen, Mathias Brekke Mandelid, Michael Reinboth, Geir Kåre Resaland, Solfrid Bratland-Sanda

**Affiliations:** ^1^ Department of Sports, Physical Education and Outdoor Studies, Faculty of Humanities, Sports and Educational Science, University of South-Eastern Norway, Bø, Norway; ^2^ Faculty of Education, Arts and Sports, Center for Physically Active Learning, Western Norway University of Applied Sciences, Sogndal, Norway; ^3^ Department of Sports, Nutrition, and Science, Faculty of Education, Arts and Sports, Western Norway University of Applied Sciences, Bergen, Norway

**Keywords:** physically active learning, physical activity, children, adolescents, school, real world

## Abstract

**Background:**

Research on physically active learning (PAL) has mainly been investigated experimentally, where interventions have been introduced to study effects on, for example, physical activity (PA) levels. This might undermine real-world contexts and realistic PA levels when teachers have sustained PAL in their regular teaching practice for several years. The purpose of this study was to observe and describe the organization and content of a variety of teaching where PAL was enacted by experienced teachers and to describe the corresponding PA levels and PA intensity in real-world practices.

**Methods:**

Fifty-eight pupils and four teachers from one primary school and one secondary school were enrolled across the first, sixth, and eighth grades. The pupils’ physical activity (PA) levels were assessed during 37 enacted physically active learning (PAL) segments within longer teaching lessons using waist-worn triaxial accelerometers. Evenson cut-off points were employed to define PA intensities. All enacted PAL were passively observed by the same observer regarding primary bodily movement, duration, subject, location, collaborative or individual work, and task orientation.

**Results:**

On average, PAL consisted of 57.8% ± 15.7% sedentary time, 22.9% ± 7.5% light intensity PA, and 19.3% ± 10.8% moderate-to-vigorous intensity PA, displaying a large variety in different PAL segments. More intensive and higher volumes of PA were evident when PAL was facilitated outdoors, in gyms, or in large stairways, while lower PA volumes and intensity were assessed when PAL was enacted inside the classroom. The primary movement in PAL was mainly running and/or walking (78.3%), while the PAL activity was mainly organized as group work (83.7%) in non-competitive tasks (97.3%).

**Conclusions:**

This study provides novel insights by being the first to investigate the organization and content of PAL enacted by experienced teachers in their teaching and the corresponding PA levels in their real-world practices. The results displayed a large diversity in PA levels and intensities and may serve as a starting point to further investigate the coherency of PA levels and PAL content in schools with sustained PAL teaching.

## 1 Introduction

Integrating physical activity (PA) into the curriculum and teaching in schools is defined as physically active learning (PAL) (1). Interventions have reported increased PA levels in pupils, with average values ranging from an additional 1 to 14 min of moderate-to-vigorous PA (MVPA) (2–8). In addition, positive effects have been reported on academic performance, mental health and vitality, and cognitive skills (1, 2, 4, 9, 10). PAL is, therefore, viewed as an important method for providing opportunities for pupils to accumulate the recommended average amount of 60 min/day of MVPA for children and adolescents advocated by the World Health Organization (WHO) (11). However, such interventions have been criticized for having limited sustainability beyond the experimental program due to poor theoretical framing, weak implementation, low adherence among teachers, and/or limited attention to real-world contexts (12, 13). Real-world contexts are here understood as the everyday practice of teaching where teachers organize their teaching according to their own purpose, values, and beliefs (14).

Against the expanding body of PAL research in educational contexts, experimental studies have been the more prevalent forms of study design compared to real-world observational studies (13). Experimental studies can be characterized as interventions designed primarily by researchers to increase PA opportunities through PAL and study the effects of specific interventions. Real-world studies, on the other hand, may be characterized as observations of PAL teaching enacted by experienced teachers who have sustained PAL as a regular part of their teaching practice (14). This may take into consideration teachers' perspectives in designing and sustaining PAL in their everyday teaching practice without scientific instructions from researchers (12, 13). Utilizing experiences from teachers who have sustained PAL in their practice beyond experimental programs is valuable to gain knowledge of PAL enactment and the corresponding PA levels in real-world practices (14, 15).

A large majority of interventions that have implemented PAL and assessed PA levels have been researcher-led, primarily with a strict randomized controlled trial design (13). Vazou et al. (13) highlighted that researcher-led interventions do not necessarily reflect a real-world context and restrict the autonomy of teachers to facilitate PAL. Furthermore, these studies may undermine the particular school context and several barriers among the teachers (e.g., lack of time and perceived value and competence of PAL enactment) (16–21). Recent studies also highlight that the primary focus of the teachers is the academic content and learning processes, rather than the amount and intensity of PA (16, 17). The coherency of academic content, PAL enactment, and PA levels and intensity has gained less attention in the previous literature, especially among teachers who have sustained PAL in their practice (12). Findings drawn from experimental studies may, therefore, not necessarily reflect pupils’ PA levels and intensities during PAL that are applicable and sustainable in a real-world context (12, 13).

There is an increasing body of knowledge on teachers' perceptions and experiences with enacting PAL in real-world teaching (17–19). However, to the best of our knowledge, no previous studies have examined the relationship between how the academic content, organization, and PA levels might look in real-world enactment of PAL in teaching. Since several PA programs in schools, including PAL, have proved ineffective (8, 10, 12, 22, 23), and teachers repeatedly have reported a greater concern for learning outcomes compared to PA levels (16–19), it is important to explore how PAL is enacted in the real world, focusing on the academic content, organization and PA levels, in schools with long experience with PAL enactment. In-depth investigations of experienced PAL teachers in case studies might provide a broad understanding of how PAL may be enacted when sustained in real-world practices. Such case studies can provide new perspectives and refine our current understanding of PAL enactment by taking into account the pedagogical perspectives of studied teachers and the specific school context (24). Knowledge derived from such studies may help guide future research designs for providing sustainable PAL programs and PA promotion in schools.

Against this background, in-depth investigations of experienced teachers and their enactment of PAL may contribute to novel and important insights into how academic content, PAL organization, and PA levels and intensity are related. Thus, this case study aimed to observe and describe the organization and content of a variety of PAL teaching situations and describe the corresponding PA levels when experienced teachers enacted PAL in their real-world practice.

## 2 Methods

### 2.1 Design

This study was an observational and descriptive case study designed to explore PAL enactment, through its academic content, organization, and PA levels. In the context of this study, PAL is understood as PA (games, drills, or other PA) integrated with academic content to teach factual information (1). Thus, this study does not include classroom movement breaks, which are ∼5 min bursts of high-intensity PA (often without academic content) enacted between academic instructions (1). The teachers recruited to the study had sustained PAL in their daily teaching after completion of a specialized 1-year continuing professional development program (CPD) in PAL teaching facilitated by the Center for Physically Active Learning (SEFAL) at the Western Norway University of Applied Sciences (17). All teachers planned and organized PAL in line with the current curriculum and their own pedagogical choices, without any instructions or guidelines from researchers. All PAL teaching was observed and objectively assessed by accelerometers by the first author (J-MJ) during three separate periods of two weeks over the course of the school year (autumn, winter, and spring). The same teachers and pupils were measured and observed during multiple lessons where PAL was used as a teaching method in the whole lesson or in specific segments of the overall lesson, to ensure a variety in PAL enactment.

### 2.2 Participants

Three different classes (first, sixth, and eighth grade) encompassing a total of 58 pupils (32 boys and 26 girls) were recruited for this study. In addition, two teachers were recruited from a primary school and two teachers were recruited from one lower-secondary school from an urban municipality located on the southeastern coastline of Norway. The pupils' characteristics are presented in Table 1. The four teachers recruited, all had formal PAL teaching education (completed CPD program), had sustained PAL in their teaching practice beyond researcher- and municipality-led experimental studies 3–4 years prior to this study, had long experience (>4 four years) with PAL enactment and a willingness to cooperate with the study. The four teachers taught different classes (one female teacher in 1st grade, one male teacher in 6th grade, and two female teachers in 8th grade) and enacted PAL two to four times a week for 4 years. The female first grade teacher primarily taught math, science, language, and music. The male sixth grade teacher taught primarily math, science, language, and physical education. The first female eighth grade teacher was a head teacher and taught math and physical education, while the other eighth grade teacher primarily taught language (English).

**Table 1:** Subject characteristics.

	All pooled			First grade			Sixth grade			Eighth grade		
	Mean	SD	95% CI	Mean	SD	95% CI	Mean	SD	95% CI	Mean	SD	95% CI
BW (kg)	43.2	17.2	38.7–47.7	24.3	4.8	22.0–26.7	45.5	7.7	41.4–49.6	56.6	14.3	50.6–62.6
BH (cm)	146.1	19.1	141.1–151.0	121.3	6.8	118.0–124.6	151.4	6.3	148.1–154.8	162.0	8.2	158.6–165.5
BMI	19.4	3.9	18.3–20.4	16.4	1.9	15.5–17.3	19.8	3.1	18.2–21.5	21.4	4.2	18.3–20.4

Values are mean, standard deviation (SD), and 95% confidence intervals (CI). BW, body weight; BH, body height; BMI, body mass index.

All teachers, pupils, and parents were given oral and written information about the nature of the study, and parental, pupil, and teacher written consent was obtained prior to data collection. All children were given information about the study adjusted for their level of understanding by their teachers and researchers. The present study protocol was evaluated and approved by the Norwegian Center for Research Data (reference number: 796490) and conducted in accordance with the Declaration of Helsinki.

### 2.3 Accelerometer assessments

Assessments of sedentary time (SED) and PA were performed by ActiGraph GT9X triaxial accelerometers (ActiGraph, LLC, Pensacola, FL, USA). In line with the objectives of the study, PA assessments were only made in lessons including PAL enactment. In all lessons including PAL teaching segments, the accelerometers were attached to the right hip with an adjustable elastic belt. All participants were instructed on how to wear the accelerometer, and the first author provided help to ensure that the accelerometers were correctly worn throughout all lessons.

The accelerometers were initialized to capture PA data with a sampling rate of 30 Hz (25) and start assessments of PA at the start of the overall lesson where the teacher had planned to enact PAL. The lessons were organized in 45- or 90-min sessions, where PAL was often used in various segments of the lesson. The pupils were equipped with accelerometers at the start of every lesson and worn throughout both traditional sedentary teaching segments and PAL segments. The accelerometers were then detached immediately after every lesson. The point where PAL was initiated was defined as the point where the teacher started PAL by giving instructions to the pupils about the provided task or instructing the pupils to move to the desired location where PAL was conducted. The termination of PAL was defined as either the point where the teacher returned to traditional sedentary teaching, pupils, and teacher returned to the classroom, or the termination of the overall lesson. After every lesson, all PA files were downloaded from the accelerometers for further analysis.

The initialization of the accelerometers and downloading and analyzing the PA files were conducted using the Actilife software package (Actigraph, LLC, Pensacola, FL, USA). File data were analyzed in 1-s epochs, since shorter epochs are recommended for the analysis of PA data in children and adolescents to especially capture high-intensity PA correctly (26, 27). For evaluating time spent in the different exercise intensity zones, Evenson cut points [0–99, 100–2, 295, 2,296–4,011, ≥4,012, and ≥2,296 cpm for sedentary time (SED), light intensity PA (LPA), moderate intensity PA (MPA), vigorous intensity PA (VPA) and MVPA, respectively] were used (28). Since an observer directly observed that pupils wore the accelerometers throughout all lessons, wear-time validation procedures were not necessary. However, all PA files were controlled for errors and potential invalid assessments. To analyze PA only in the part where PAL was used, the time filtering option in the software program was used to filter out only the PAL segments.

### 2.4 Observational data

Descriptions of the academic content and organization of PAL were obtained through direct observations and field notes (in a standardized form and unstructured notes) by the first author. The first author was present at the school throughout the whole day and followed the teachers in other school lectures and breaks as well. This allowed the researchers to collaborate closely with the teachers and build a trusting relationship. This provided a foundation for discussing, reflecting on, and elaborating upon the PAL activity and the observations and field notes closely with the teachers before and after every PAL segment to better interpret the data and to ensure consistency between observations.

Sedentary teaching prior to or after PAL was not observed, although the observer was present at these parts as well. All observations were manually written and described in field notes. Observational data for the following categories were collected in all PAL segments of the lesson: exact duration, location, subject, primary bodily movement, collaborative or individual work, and task orientation (competitive vs. non-competitive). The nature and description of the academic task provided by the teachers were written down in field notes.

The exact time of the start and end of each PAL was registered manually and was defined identically as the accelerometer assessments described above. The location where PAL was organized was defined as either outdoor, indoor, or in classroom. Outdoor was defined as all PAL enacted in the schoolyard or nearby environment (e.g., woods and soccer field). Indoor was defined as all PAL facilitated inside the school buildings but outside of the classroom (e.g., stairways and gym). Classroom was defined as all PAL enacted inside the classroom. Subject types were categorized according to the current curriculum and regular school subjects (e.g., mathematics, language, and science). Primary bodily movement was defined as the movement that was used by the pupils for the majority of the time during PAL and was registered as either running, walking, throwing, jumping, strength exercises, crawling, climbing, or combinations of movements (i.e., running and throwing in ball games). These movement categories were drawn from the framework provided by Hulteen et al. (29) on foundational movement skills. Collaborative or individual work was defined as activities where the pupils were organized individually, in pairs, or in larger groups (>2 pupils) to work on the academic task. The task orientation category was defined as whether the academic task was organized as competitive or not. To determine if the task was competitive, the teacher had to instruct the pupils to compete with other pupils (e.g., solve a specific task the fastest, and get the most points in a specific task). Any uncertainties surrounding the observations were discussed with the teachers for confirmation or potential adjustments.

### 2.5 Statistics

Normality tests (Kolmogorov–Smirnov) and Q–Q plots were performed to determine if the data showed a normal distribution. Some variables did show a normal distribution (SED and LPA), although some variables did not (MPA, VPA, and MVPA). Parametric and non-parametric statistics were compared, and similar results were displayed. Thus, parametric statistics were used to present the data. Descriptive statistics were used to present data as mean ± standard deviation (SD), 95% confidence intervals (CI), variation coefficient (VC), and range. All observational data are presented as frequencies and percentages of the whole sample of individual PAL overall and within grade levels. Due to the nature of the study and the small sample size, no further parametric analyses were conducted. Statements of any differences in PA levels between enacted PAL segments, grade levels, PAL locations, and more were made solely by reviewing descriptive results. Statistical Package of Social Sciences (SPSS) version 29.1 was used for all statistical analyses.

## 3 Results

In total, structured observations and objectively assessed PA were obtained from 37 school lessons where PAL was enacted in teaching (first grade, 12 lessons; sixth grade, 15 lessons; eighth grade, 10 lessons). The teachers planned and performed PAL 2–3 times per week, in subjects related to Norwegian language (*n* = 8), English language (*n* = 13), and mathematics (*n* = 16). PAL was typically enacted as a segment and combined with more traditional, sedentary, and classroom-based teaching. However, in three instances, PAL was enacted as a whole lesson (±45 min) (one in first grade and two in sixth grade; see Supplementary Tables 1 and 2).

**Figure 1:**
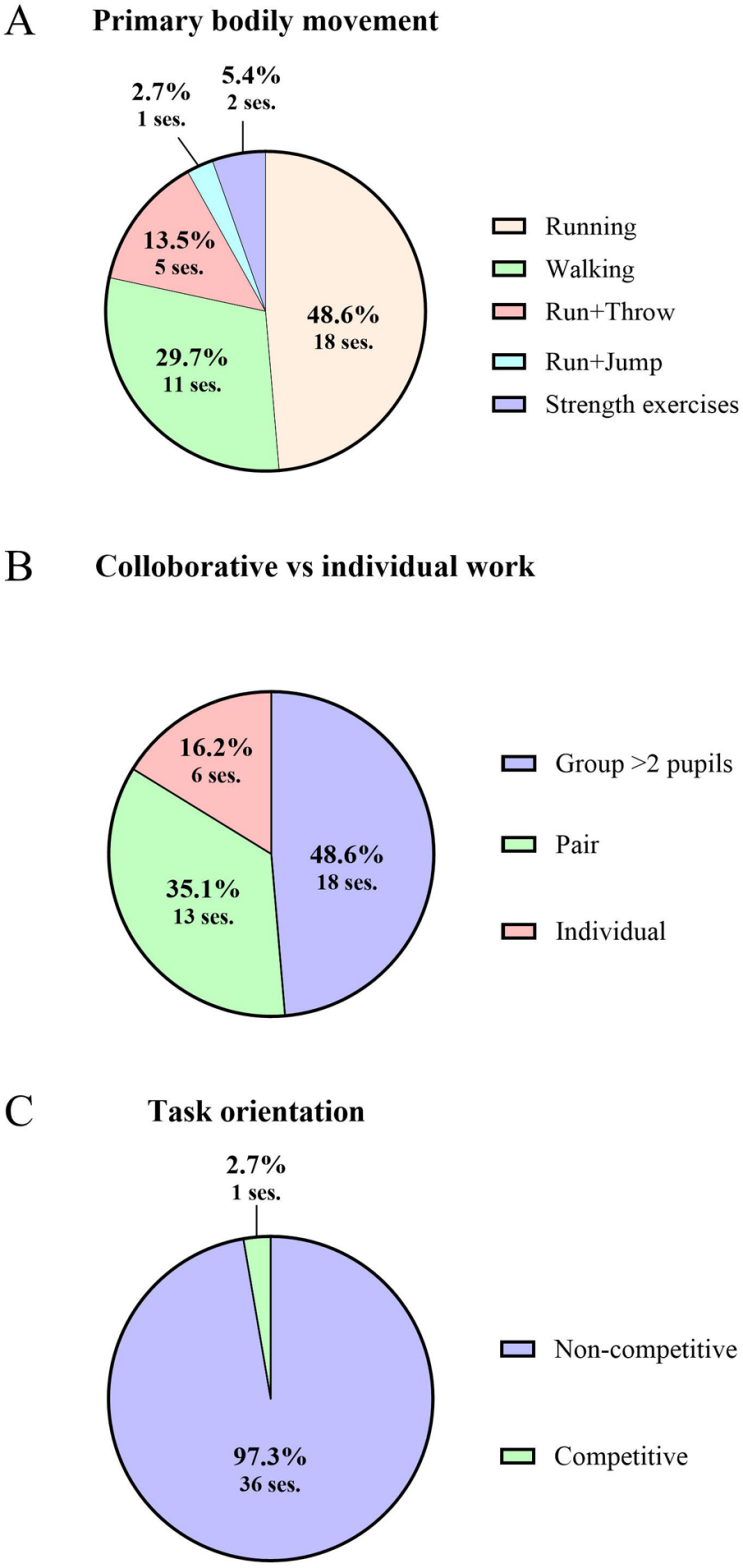
Overview of the organization of PAL enacted in teaching. The figure displays the **(A)** frequency and percentage of primary bodily movement used by pupils in observed PAL, **(B)** frequency and percentage of PAL organized as collaborative-, pair-, or individual work, and **(C)** frequency and percentage of PAL organized with competitive- or non-competitive academic task.

Observations of enacted PAL are summarized in Figures 1A–C. The primary movement selected by the teacher was mainly running or walking (Figure 1A). The two segments of PAL that were categorized with bodily movements related to different strength exercises, were both observed in 8th grade. Generally, the percentage of time spent in MVPA and total PA was 12.2% (SE = 0.8, 95% CI = 10.7, 13.9) and 17.9% (SE = 1.4, 95% CI = 15.2, 20.6) higher, respectively, and 15.8% (SE = 1.4, 95% CI = 13.1, 18.4) lower percentages of SED in PAL that included running compared to PAL where walking was a primary movement (Supplementary Tables 1–3). Primarily, the teachers also organized tasks and PAL activities as group work (83.7%, Figure 1B) and did not provide competitive instructions in most PAL teaching (Figure 1C). The only session that was organized as a competitive game intentionally by the teacher was conducted in first grade.

The general characteristics of PA in PAL are presented in Table 2. On average, PAL segments generated 9.8 ± 4.0 min of total PA (LPA + MVPA) ranging from 0.1 to 26.9 min. Generally, PAL segments were mainly dominated by SED (57.8% ± 15.7%; range: 18.8%–99.8%), while LPA, MPA, and VPA contributed with 22.9% ± 7.5% (range: 0.1%–45.9%), 8.1% ± 4.6% (range: 0.0%–27.9%), and 11.1 ± 7.3% (range: 0.0%–43.5%) of each PAL segment, respectively.

**Table 2:** Physical activity and duration characteristics in PAL.

	All pooled			First grade			Sixth grade			Eighth grade		
	Mean	SD	VC (%)	Mean	SD	VC (%)	Mean	SD	VC (%)	Mean	SD	VC (%)
Duration												
Min	22.2	7.2	32.4	19.4	5.8	29.9	27.4	7.9	28.8	20.8	5.7	27.4
PA characteristics												
SED												
Min	14.2	7.1	50.0	12.9	7.6	58.9	17.6	7.3	41.5	12.3	5.1	41.5
%	57.8	15.7	27.2	54.9	15.0	27.3	62.9	16.9	26.9	55.9	13.9	24.9
LPA												
Min	5.4	2.2	40.7	5.4	2.2	40.7	5.6	2.2	39.3	5.3	2.4	45.3
%	22.9	7.5	32.8	24.5	7.4	30.2	21.3	8.2	38.5	23.0	6.7	29.1
MPA												
Min	1.8	1.1	61.1	1.7	0.7	41.2	1.9	1.2	63.2	2.0	1.2	60.0
%	8.1	4.6	56.8	8.2	3.8	46.3	7.2	4.8	66.7	9.0	4.9	54.4
VPA												
Min	2.5	1.7	68.0	2.5	1.3	52.0	2.4	2.0	83.3	2.6	1.6	61.5
%	11.1	7.3	65.8	12.5	7.4	59.2	8.9	7.3	82.0	12.1	6.8	56.2
MVPA												
Min	4.3	2.5	58.1	4.2	1.9	45.2	4.2	3.0	71.4	4.5	2.5	55.6
%	19.3	10.8	56.0	20.7	10.3	49.8	16.0	11.2	70.0	21.0	10.3	49.0

Values are mean, standard deviation (SD), and variation coefficient (VC) in percentage. cpm, counts per minute. SED, sedentary behavior. LPA, light physical activity; MPA, moderate physical activity; VPA, vigorous physical activity; MVPA, moderate-to-vigorous physical activity; Min, minutes per PAL session; %, percent.

There were negligible differences between classes in total PA in minutes (9.7 ± 3.3 min, 9.9 ± 4.6 min, and 9.8 ± 3.9 min for the first, sixth, and eighth grade, respectively) or in the number of minutes spent in the different PA intensities (LPA, MPA, VPA, or MVPA; Table 2). However, the sixth grade displayed higher amounts of SED (+8.0 and +7.0%, +4.7 and +5.3 min) and a lower percentage of LPA (−3.2% and −1.7%) and MVPA (−4.7% and −5.0%) compared to the first and eighth grade, respectively (Table 2).

Table 3 presents the descriptive information on PA characteristics during PAL conducted in different locations (outdoor, indoor, and inside the classroom). In general, classroom-based PAL generated higher relative values of SED (+11.5% and +9.4% higher compared to indoor and outdoor PAL, respectively) both when the data were pooled and within each class (Table 3). Indoor-based (+8.9%) and/or outdoor-based (6.9%) PAL generated more PA at higher intensities (i.e., MVPA) compared to classroom-based PAL.

**Table 3:** Descriptive data on physical activity levels in different PAL locations.

	Location	SED		LPA		MVPA		Total PA	
		Min	%	Min	%	Min	%	Min	%
	Outdoor (*n* = 12)	14.4 ± 7.4	55.6 ± 15.7	6.0 ± 2.6	23.5 ± 8.2	5.1 ± 3.0	20.7 ± 10.6	11.1 ± 4.6	45.7 ± 16.9
All	Indoor (*n* = 12)	11.7 ± 5.7	53.5 ± 14.4	5.0 ± 1.6	23.9 ± 6.2	4.6 ± 2.1	22.7 ± 10.4	9.6 ± 3.1	46.7 ± 14.3
	Classroom (*n* = 12)	16.8 ± 7.4	65.0 ± 14.7	5.3 ± 2.3	21.2 ± 7.9	3.2 ± 1.9	13.8 ± 9.4	8.4 ± 3.6	37.8 ± 16.9
First grade	Outdoor (*n* = 4)	9.6 ± 2.7	55.3 ± 15.9	4.4 ± 1.8	24.3 ± 8.3	3.6 ± 1.9	20.4 ± 10.3	8.0 ± 3.4	44.7 ± 16.9
	Indoor (*n* = 4)	9.4 ± 2.5	50.1 ± 11.0	4.6 ± 1.2	24.7 ± 5.6	4.7 ± 1.5	25.2 ± 7.4	9.3 ± 2.3	49.9 ± 11.0
	Classroom (*n* = 4)	19.6 ± 9.5	59.0 ± 16.2	7.3 ± 2.1	24.4 ± 7.9	4.5 ± 2.0	16.6 ± 11.5	11.7 ± 2.9	48.6 ± 21.6
Sixth grade	Outdoor (*n* = 4)	17.7 ± 10.6	49.4 ± 16.7	7.7 ± 2.1	25.3 ± 8.6	7.7 ± 3.2	25.3 ± 12.0	15.4 ± 4.1	50.6 ± 16.7
	Indoor (*n* = 5)	17.4 ± 6.4	64.7 ± 12.9	5.2 ± 1.4	20.6 ± 6.1	3.7 ± 1.4	15.1 ± 8.7	8.8 ± 2.3	36.0 ± 13.1
	Classroom (*n* = 6)	17.7 ± 5.1	70.7 ± 14.4	4.6 ± 1.8	19.2 ± 8.5	2.3 ± 1.6	10.2 ± 7.9	6.9 ± 2.8	29.3 ± 14.4
Eighth grade	Outdoor (*n* = 4)	16.3 ± 5.3	60.1 ± 13.3	6.4 ± 2.6	21.6 ± 7.5	4.7 ± 2.5	18.0 ± 9.4	11.1 ± 3.5	43.4 ± 17.4
	Indoor (*n* = 4)	9.0 ± 2.3	46.8 ± 12.5	5.2 ± 2.0	26.1 ± 5.7	5.4 ± 2.6	27.1 ± 10.4	10.6 ± 4.0	53.2 ± 12.5
	Classroom (*n* = 2)	10.7 ± 1.4	64.3 ± 6.4	3.3 ± 0.8	19.8 ± 4.5	2.7 ± 1.0	15.9 ± 5.4	6.0 ± 1.3	35.7 ± 6.4

Values are mean ± standard deviation expressed in minutes and percentages. SED, sedentary time; LPA, light intensity physical activity; MVPA, moderate-to-vigorous intensity physical activity; total PA, total amount of physical activity based on values from LPA and MVPA.

The large variation coefficients presented in Table 2 reveal a great variation in PA levels across PAL. To depict the variation between lessons, PAL with the highest and lowest amount of PA and intensity are presented with corresponding duration, primary bodily movement, location, subject, and academic task in Table 4. All PAL segments observed and assessed are presented in the same way in Supplementary Tables 1–3. Table 4 and the supplementary files display a large diversity in PAL delivered by the teachers in terms of PA level, PA intensity, duration, and different organizational aspects.

**Table 4:** Description of PAL sessions with the highest and lowest amount of PA.

Duration	PA level	PA characteristics	Primary movement	Subject	Location	Academic task
First grade						
20 min	High	SED: 7.7 min (38.5%)	Running and fast walking	Mathematics	Classroom	Topic: shapes and geometry. Boxes were placed at one end of the classroom with figures of triangles, squares, rectangles, etc. Individually, pupils were instructed to pick up one figure at a time and sort as many as possible into corresponding boxes at the other end of the classroom.
		LPA: 5.7 min (28.4%)				
		MVPA: 6.6 min (33.1%)				
		Total PA: 12.3 min (61.5%)				
41 min	Low	SED: 31.7 min (77.3%)	Walking	Norwegian (language)	Classroom	Topic: letters and words. Each pupil was instructed to walk up to the blackboard and pick up a letter from a box. Afterward, they were supposed to walk down to their desk and write a word on their computer that started with this letter.
		LPA: 6.6 min (16.2%)				
		MVPA: 2.7 min (6.5%)				
		Total PA: 9.3 min (22.7%)				
Sixth grade						
26 min	High	SED: 8.2 min (31.6%)	Running	Mathematics	Outdoor	Topic: repetition of plus/minus. Tiles with numbers from 0 to 100 were spread out on a field. Using mental arithmetic and throwing a dice (0–10), groups of pupils were instructed to move from tile to tile according to the previous number plus the number on the dice up to 100. Then the same exercise by subtracting the dice number down to 0.
		LPA: 8.0 min (30.9%)				
		MVPA: 9.7 min (37.4%)				
		Total PA: 17.9 min (68.4%)				
26 min	Low	SED: 21.8 min (83.9%)	Walking	Mathematics	Classroom	Topic: fraction calculation. The teacher had put up different mathematical tasks on the walls. In pairs, pupils were instructed to move around to solve these tasks by discussing possible solutions with each other, while the teacher provided supervision.
		LPA: 3.2 min (12.3%)				
		MVPA: 1.0 min (3.8%)				
		Total PA: 4.2 min (16.1%)				
Eighth grade						
28 min	High	SED: 11.8 min (42.1%)	Running	English (language)	Indoor (gym)	Roald Dahl. Pupils played a ball game in the gym, where they threw soft balls at each other. Those that were hit were given penalty exercises (five squats). Now and then the teacher stopped the game to give the pupils factual sentences about Roald Dahl that they had to retell to another pupil who was supposed to write it on a paper. After that, they returned to the game.
		LPA: 8.1 min (29.1%)				
		MVPA: 8.1 min (28.9%)				
		Total PA: 16.2 min (58.0%)				
15 min	Low	SED: 9.7 min (64.6%)	Strength exercises	Mathematics	Classroom	Topic: individual work with mathematical tasks. All pupils worked at their desks with mathematical tasks. Every 10 min the teacher stopped the work, and the pupils followed four short videos (2–7 min each) with physical exercises together with the teacher.
		LPA: 3.1 min (20.5%)				
		MVPA: 2.2 min (14.8%)				
		Total PA: 5.3 min (35.4%)				

Values of physical activity are presented as mean and percentage of the PAL session in parentheses. PA, physical activity; SED, sedentary time; LPA, light physical activity; MVPA, moderate-to-vigorous physical activity.

## 4 Discussions

This study breaks new ground by being the first to observe PAL enactment and academic content and describe the corresponding PA levels when experienced teachers enact PAL in their real-world practice. The findings contribute to our understanding of how different ways of enacting PAL may contribute to the amounts of total PA and MVPA among children and adolescents in a day-to-day setting. In addition, this study provides novel perspectives into how the coherency of academic content and organization of PAL influence PA levels and intensity. Overall, the main findings displayed a large diversity in total PA levels and intensity distribution during PAL. Teachers mainly organized PAL as non-competitive group work tasks. Most of the time, higher PA intensities were generally generated through PAL facilitated outside of the classroom. Running and/or walking was, in most cases, selected as the primary movement.

The PA levels during PAL were comparable to what Morris et al. (30) and Vazou et al. (7) reported from their experimental studies. However, the results from this study are lower than what has been reported from other experimental studies and systematic reviews examining the effect of PAL on PA levels (3, 5, 6, 8). However, it is challenging to compare the PA data from the present study to other experimental studies due to the differences in study design (experimental study vs. real-world case study). Although the sample size in the present study was small, our results might suggest that when teachers enact PAL in their real-world practice, PA levels might be lower compared to researcher-led, experimental studies. On the other hand, some PAL segments observed in the present study included high amounts of total PA and MVPA, but this was evident when teachers made sense of it in a pedagogical and didactical context. The present results might be supported by recent qualitative PAL studies reporting that teachers are more concerned with learning processes and academic outcomes rather than the sole health perspective and PA levels (16–18). Moreover, evidence suggests that the desired educational purposes guide how PAL is enacted and thus the corresponding PA intensity and PA level (18, 19). This may be illustrated in Table 4 and the two examples from sixth grade. The two sessions were of equal duration, led by the same teacher and in the same subject, yet with different planned academic tasks and curricular learning outcomes. This led to different content and organization and a large difference in PA levels (1.0 min vs. 9.7 min of MVPA; Table 4). The findings of Mandelid et al. (18) resonate with the present findings that more dialogical and collaborative tasks generate different PA levels and intensity compared to tasks organized purely for repetition purposes.

The nuanced perspective of teachers on PA levels in PAL enactment reported in previous studies (16–18) and the aforementioned perspectives may also be reflected by the present teachers organizing the majority of PAL (83.7%) as non-competitive, collaborative group (>2 pupils) or pair work. Teslo et al. (17) and Schmidt et al. (31) reported that teachers and pupils embrace this way of organizing PAL because it is perceived as an important contributor to a more inclusive learning environment. In the same vein, the findings of Schmidt et al. (32) highlighted that teachers wanted to avoid creating competitive activities to ensure that all pupils were included, although some pupils often seem to have a competitive mindset regardless of how little the teachers emphasize it (31, 33). Previous studies have also reported that PAL enacted in teaching, to a larger degree than traditional classroom teaching, creates opportunities to reach social goals in school (16, 17). In a Norwegian educational context, the social perspective of PAL also adheres to the school's goal of developing and educating children with life skills to become active and engaged citizens (34, 35). Such perspectives might influence what PA levels are most suitable for PAL enactment and what PA levels can be expected in real-world contexts. As the present study indicates, the teachers' perspectives and freedom to organize PAL should be accounted for when evaluating PA levels. Hence, we argue that the results from the present study may reflect PA levels generated through PAL that might be expected when teachers make sense of enacting PAL and are thus more applicable and sustainable in a real-world context.

Although SED generally dominated PAL, approximately 10 min of total PA were generated on average. Considering that teachers generally enacted PAL 2–3 times per week, this implies 20–30 min of additional PA per week, with 10–15 min categorized as MVPA (Table 2). Although the present study includes a small sample of pupils and teachers, the results might indicate that children exposed to PAL weekly throughout 10 years in primary and secondary schools will be able to accumulate a valuable amount of PA that they might not otherwise have done. In addition, the everyday encounter with PA in school, either at higher or lower intensities, may shape positive attitudes toward PA and, therefore, be an important contributor to public health. Future studies should investigate these potential benefits in more detail. This is also interesting in relation to the WHO adjusting their recommendations in 2020 to emphasize that “every move count” (11), which implies that all PA, even at lower intensities, becomes important.

In terms of MVPA levels, the contribution from PAL in the present study was approximately 7% of the recommended 60 min of daily MVPA recommended by WHO for children and adolescents (11). This is somewhat lower compared to previous experimental studies (4–6, 8). On the other hand, some enacted PAL generated almost 30% of the recommended MVPA levels in individual pupils (Table 4). The present study, therefore, provides novel insights into not only the variety of PA intensity in PAL enacted in different teaching lessons by experienced teachers but also how much MVPA and total PA can be expected through this teaching method. In addition, PAL facilitated outdoors or inside in stairways and gyms generated more MVPA compared to PAL in the classroom. This is in line with previous studies that have reported that moving pupils outdoors during various school activities positively affects total PA and MVPA levels (36–38). From a health perspective, moving PAL out of the classroom may have a higher potential for incorporating more intensive PA in PAL and, therefore, contribute to the WHO guidelines to a greater extent (11). However, as the present results display, moving PAL outdoors for more intensive PA is conducted when teachers make sense of that with regard to the academic task and preferred learning outcome.

All primary movements observed in PAL corresponded to foundational movement skills, as defined by Hulteen et al. (29). The observed teachers organized their PAL mainly by use of familiar movement patterns, such as running (48.6%) and walking (29.7%). This corresponds with findings of movement patterns of pupils during recess in school (39). However, there needs to be more knowledge of which movement patterns are predominantly used in teacher-led PA, such as PAL and physical education. In a previous meta-analysis, the rate of participation in foundational movement skills (e.g., running, jumping, and strength exercises) was associated with higher levels of PA throughout the lifespan (40). Therefore, PAL may be an important tool to ensure that children and adolescents develop and participate in more foundational movements throughout early life and create a foundation to sustain higher PA levels throughout the lifespan.

### 4.1 Implications and practical recommendations

The present study provides novel insights that can serve as a starting point for future research to explore the relationship between teachers’ PAL enactment in real-world practices, the academic content, and the PA levels. This study, therefore, calls upon comparable studies of similarly competent and experienced PAL teachers observed in their real-world practice. In this way, our understanding of how sustained PAL enactment might look and how much PA (both in volume and intensity) can be expected in pupils through PAL can be extended beyond the present study. This may also provide knowledge of how PAL as a sustained part of teachers’ teaching practice may serve as a sustainable PA and health promoter.

The results presented might also serve as inspiration for other teachers struggling to sustain PAL as a natural part of their everyday practice. This study provides examples of how experienced teachers enact PAL purposefully in their pedagogical practice, which can provide new perspectives and pedagogical reflections in other teachers. The diversity of PA levels and intensity among PAL segments in this study might provide novel perspectives on the coherency of academic content and the volume and intensity of PA that are purposeful to support the learning outcome. This can, therefore, help accommodate some of the perceived barriers to enact and sustain PAL in other teachers (16–21, 41).

We recommend that teachers carefully consider where to integrate PA to enhance learning and how to do this purposefully. This might result in various PA levels in completely different activities, as seen in the examples provided in Table 4. As this study indicates that PAL can vary greatly in PA levels, teachers should be careful to evaluate the success of PAL solely based on health and PA objectives nor utilize a set amount of MVPA in everyday enactment. Instead, teachers should evaluate the degree of success based on pedagogical perspectives and the purposes that they set out to achieve. We do not recommend a “one-PAL-fits-all” approach, especially when PAL is enacted in different grade levels. Enacting PAL for primary school pupils might be different from enacting PAL for lower-secondary school pupils, due to differences in, e.g., academic pressure and assessments. Since PA levels have been shown to decrease with age (42), this also highlights the importance of PAL in secondary schools.

Furthermore, we also recommend that the present study can serve as a departure point to help guide research in the development of more sustainable programs for PAL enactment in schools. Case studies, such as the present study, might provide important perspectives, nuances, and contextual factors that are often lost in larger experimental studies (24). Increased knowledge of such factors may provide researchers the ability to design studies that are suitable for schools in the real world. Therefore, learning from teachers with long experience of PAL enactment might be valuable both for teachers considering if they should implement PAL and how to do this in their teaching practice, and for researchers in their development of future study designs.

Since MVPA levels from the present study were somewhat lower than that reported in most experimental studies (3, 5, 6, 8), this confirms the recommendations that PAL should be viewed as a part of a whole-school approach for increasing PA in children and adolescents (12). Since whole-school PA programs often have proved ineffective and difficult to sustain (12), we recommend that studies with a real-world approach, such as the present study, may serve as a departure point for designing more targeted and sustainable research designs for overall PA promotion in schools. In addition, to be able to reach the WHO recommendations (11), sustainable PA promotion in activities outside of school as well should also be given more emphasis in future investigations (23).

### 4.2 Limitations

The major limitation of the present study is the small number of schools, teachers, and pupils recruited. Therefore, generalization of the present results to other schools is not enabled. Similar studies have to be conducted before any general statements about the coherency of academic content and enactment of PAL and the corresponding PA levels in real-world contexts can be made. We acknowledge that this study only presents how PAL was enacted and how this influenced PA levels in these three classes at two different schools in a Norwegian school context and that the results might have looked different if other classes had been investigated. Investigations similar to the present study of schools in other regions of Norway or other countries may not reveal similar results, due to different teachers, pupils, environments, and school policies. Since the present data only provides information on how PAL was enacted and how much PA was generated through PAL in these three classes, interpretation should be cautious. Another limitation is that differences in PA levels between grade levels, PAL locations, and enacted PAL were made solely by reviewing descriptive results. Therefore, statements of whether these differences are statistically significant are not possible. Future studies should emphasize a similar study approach as the present study in larger sample sizes. In this way, the present matter could also be investigated statistically.

The number of enacted PAL observed in the present study still provides a solid foundation to describe and discuss the variation in how PAL was organized and the corresponding PA levels in the respective schools that were investigated. The combination of accelerometer-assessed PA levels and the structured observations and discussions with the responsible teachers enabled a thorough exploration of PAL in a real-world context.

## 5 Conclusions

When experienced PAL teachers enacted PAL in their real-world practice, a great coherency was observed between academic content, PAL organization, and levels and intensity of PA. Teachers enacted PAL with great emphasis on pupils working in groups within tasks without competitive instructions and with mainly running or walking as the primary movement patterns. Although SED dominated PAL, MVPA levels ranged from negligible to almost one-third of the duration. Accordingly, the intensity and levels of PA seemed to be influenced by how teachers organized PAL activities in terms of location, bodily movement patterns, academic content, and collaborative work.

## Data Availability

The original contributions presented in the study are included in the article/Supplementary Material; further inquiries can be directed to the corresponding author.
