# Supplementary material for: Physical activity and content in a variety of physically active learning: an observational case study of real-world practices
Source: Front Sports Act Living. 2025 Jan 3;6:1504704. doi: 10.3389/fspor.2024.1504704 (PMC11739032; doi:10.3389/fspor.2024.1504704)
Supplement: Supplementary file 1 [file Table1.docx]

| **Additional File 1: Description of all PAL teaching assessed and observed in the 1^st^ grade.** | | | | | | |
| --- | --- | --- | --- | --- | --- | --- |
|  | Duration | PA characteristics | Primary  movement | Subject | Location | Academic task |
|  |  | | | | | |
| 1. | 20 min | SED: 8.4 min (41.8%) | Running and  throwing | English (language) | Outdoor  (ball pit) | **English words.** Pupils played a ball game, where they threw soft balls at each other. Those that were hit, ran to the teacher. The teacher held up a picture, and the pupils were supposed to say, “I can see…” and name one thing they saw in the picture. Afterwards they engaged in the ball game until they were hit once more. |
|  |  | LPA: 6.3 min (31.3%) |  |  |  |  |
|  |  | MVPA: 5.3 min (26.9%) |  |  |  |  |
|  |  | Total PA: 11.6 min (58.2%) |  |  |  |  |
|  |  |  |  |  |  |  |
| 2. | 41 min | SED: 31.7 min (77.3%) | Walking | Norwegian  (language) | Classroom | **Letters and words.** Each pupil was instructed to walk up to the front of the classroom and pick up a letter from a box. Afterwards they were supposed to walk down to their desk and write a word on their computer that started with this letter. |
|  |  | LPA: 6.6 min (16.2%) |  |  |  |  |
|  |  | MVPA: 2.7 min (6.5%) |  |  |  |  |
|  |  | Total PA: 9.3 min (22.7%) |  |  |  |  |
|  |  | | | | | |
| 3. | 27 min | SED: 14.9 min (55.0%) | Walking | Mathematics | Classroom | **Lengths and measurements.** Pupils received a paper with pictures of 10 items in the classroom. Pupils walked around the classroom in pairs and were supposed to measure all the items pictured. Measurements were performed by placing their palms after each other and register how many that was necessary to measure the total length of each item. |
|  |  | LPA: 7.8 min (29.0%) |  |  |  |  |
|  |  | MVPA: 4.3 min (15.9%) |  |  |  |  |
|  |  | Total PA: 12.1 min (45.0%) |  |  |  |  |
|  |  |  |  |  |  |  |
| 4. | 15 min | SED: 10.8 min (71.8%) | Running | Norwegian  (language) | Outdoor  (schoolyard) | **Words and letters.** Pupils were divided in groups with a paper of different pictures. One by one were supposed to travel through a short obstacle course and pick up small pictures at the end and travel back to the group. Back at the group they were supposed to fit pictures on the paper with the collected pictures from the obstacle course based on the following example: picture on paper showing a dice with the text “Dice -D”. The collected picture that fit this would show a picture of “Ice”. |
|  |  | LPA: 2.4 min (16.2%) |  |  |  |  |
|  |  | MVPA: 1.8 min (12.0%) |  |  |  |  |
|  |  | Total PA: 4.2 min (28.2%) |  |  |  |  |
|  |  | | | | | |
| 5. | 18.5 min | SED: 9.9 min (52.3%) | Running | Norwegian  (language) | Indoor  (stairway) | **Words and syllables.** Teacher randomly put out patches with words, pictures, and syllables in a large stairway. Pupils were supposed to gather these patches and first connect words and pictures together. Afterwards they were supposed to do the similar task with connecting syllables into words. |
|  |  | LPA: 4.8 min (25.5%) |  |  |  |  |
|  |  | MVPA: 4.2 min (22.2%) |  |  |  |  |
|  |  | Total PA: 9.0 min (47.7%) |  |  |  |  |
|  |  |  |  |  |  |  |
| 6. | 19.5 min | SED: 10.6 min (54.5%) | Running | Mathematics | Indoor  (stairway) | **Number doubling.** Pupils were divided in groups with a bingo board with numbers at the bottom of a large stairway. Large dices are placed at the top, and pupils ran up and threw the dice one by one. The pupils were supposed to double the number on the dice and run down to the group and mark the corresponding number on the bingo board. |
|  |  | LPA: 4.3 min (22.3%) |  |  |  |  |
|  |  | MVPA: 4.5 min (23.3%) |  |  |  |  |
|  |  | Total PA: 8.8 min (45.6%) |  |  |  |  |
| **Additional File 1 cont.: Description of all PAL teaching assessed and observed in the 1^st^ grade.** | | | | | | |
|  | Duration | PA characteristics | Primary  movement | Subject | Location | Academic task |
|  | | | | | | |
| 7. | 15 min | SED: 7.2 min (46.5%) | Running | Mathematics | Indoor (stairway) | **Number doubling.** Pupils worked in pairs and were handed a paper with drawings of numbered boxes, a mouse, and a cheese. Pupils ran up the stairs and threw a dice, doubled the number on the dice, ran down and colored the box with the corresponding number. This was continued until the mouse had a colored “path” to the cheese. |
|  |  | LPA: 3.9 min (25.1%) |  |  |  |  |
|  |  | MVPA: 4.4 min (28.5%) |  |  |  |  |
|  |  | Total PA: 8.3 min (53.6%) |  |  |  |  |
|  | | | | | | |
| 8. | 20 min | SED: 7.7 min (38.5%) | Running | Mathematics | Classroom | **Shapes and geometry.** All pupils worked individually. Boxes were placed in one end of the classroom with figures of triangles, squares, rectangles, etc. The pupils were instructed to pick up one figure at the time and sort as many as possible into corresponding boxes at the other end of the classroom. |
|  |  | LPA: 5.7 min (28.4%) |  |  |  |  |
|  |  | MVPA: 6.6 min (33.1%) |  |  |  |  |
|  |  | Total PA: 12.3 min (61.5%) |  |  |  |  |
|  | | | | | | |
| 9. | 20.5 min | SED: 9.9 min (47.1%) | Running | Mathematics | Indoor  (stairway) | **Subtraction.** This task was organized as a competitive game. Pupils were divided into groups of 2-3 pupils, each group with a tower of 10 bricks. One pupil from each group ran up the stairs and threw a dice at the top. Then they ran down to the group and removed bricks from the tower based on the number from the dice. The pupil removing the last brick, obtained one point. The game was then repeated. |
|  |  | LPA: 5.4 min (25.9%) |  |  |  |  |
|  |  | MVPA: 5.7 min (27.0%) |  |  |  |  |
|  |  | Total PA: 11.1 min (52.9%) |  |  |  |  |
|  | | | | | | |
| 10. | 14.5 min | SED: 6.9 min (47.5%) | Running | English (language) | Outdoor  (school-yard) | **English words and weather.** Pupils were divided into groups for a memory game. One pupil from each group was running towards a group of cards with either pictures of different weather, or the word for different weather types (e.g., sunny, raining). If they found corresponding cards they returned to the group with cards, and without cards if they failed to find corresponding cards. The game was repeated several times. |
|  |  | LPA: 4.0 min (27.0%) |  |  |  |  |
|  |  | MVPA: 3.7 min (25.5%) |  |  |  |  |
|  |  | Total PA: 7.7 min (52.5%) |  |  |  |  |
|  | | | | | | |
| 11. | 21 min | SED: 12.5 min (59.4%) | Running | Norwegian (language) | Classroom | **Norwegian words.** Pupils worked in pairs and was delivered patches with a Norwegian word written on it. The task was then to run into another room and find a picture of the word they were delivered. This was then handed over to the teacher, which gave the pairs a new word. |
|  |  | LPA: 4.9 min (23.2%) |  |  |  |  |
|  |  | MVPA: 3.7 min (17.5%) |  |  |  |  |
|  |  | Total PA: 8.6 min (40.7%) |  |  |  |  |
|  | | | | | | |
| 12. | 37 min | SED: 23.2 min (62.7%) | Running | Mathematics | Outdoor  (school-yard) | **Subtraction.** Pupils were divided into groups with a drawing of eggs and chickens. One pupil from each group was running to pick up cards with numbers that were placed randomly in the schoolyard. Back at the group, pupils were supposed to color egg that was numbered according to number on the collected card minus 1. |
|  |  | LPA: 9.1 min (24.7%) |  |  |  |  |
|  |  | MVPA: 4.6 min (12.6%) |  |  |  |  |
|  |  | Total PA: 13.7 min (37.3%) |  |  |  |  |
| Values of physical activity are presented as mean and percentage of the PAL session in parentheses. PA, physical activity. SED, sedentary time, LPA, light physical activity. MVPA, moderate-to-vigorous physical activity. | | | | | | |
