# Supplementary material for: Physical activity and content in a variety of physically active learning: an observational case study of real-world practices
Source: Front Sports Act Living. 2025 Jan 3;6:1504704. doi: 10.3389/fspor.2024.1504704 (PMC11739032; doi:10.3389/fspor.2024.1504704)
Supplement: Supplementary file 2 [file Table2.docx]

| **Additional File 2: Description of all PAL teaching assessed and observed in the 6^th^ grade.** | | | | | | | | | | | | |
| --- | --- | --- | --- | --- | --- | --- | --- | --- | --- | --- | --- | --- |
|  | Duration | | PA characteristics | | Primary  movement | | Subject | | Location | | Academic task | |
|  |  | | | | | | | | | | | |
| 1. | 22 min | | SED: 13.7 min (62.3%) | | Walking | | Norwegian  (language) | | Classroom | | **Sources of literature.** Pupils worked in pairs and walked several times to a table and picked up words related to sources of literature with three alternative meanings of the word. The pupils were then supposed to discuss and find the correct meaning. This task was followed by a quick walk/run around the school building before the task was repeated, but now with the pupils explaining the words to each other. | |
|  |  |  | LPA: 4.0 min (18.1%) | |  |  |  |  |  |  |  |  |
|  |  |  | MVPA: 4.3 min (19.6%) | |  |  |  |  |  |  |  |  |
|  |  |  | Total PA: 8.3 min (37.7%) | |  |  |  |  |  |  |  |  |
|  |  | |  | |  | |  | |  | |  | |
| 2. | 27 min | | SED: 19.4 min (71.8%) | | Walking | | English  (language) | | Classroom | | **English glossary.** Pupils worked in groups and were supposed to walk and collect words according to pictures they were given by the teacher. When they were finished, they received a text with a description of a pictured item somewhere inside the classroom. Based on the text, the pupils walked and collected the correct picture. | |
|  |  |  | LPA: 5.5 min (20.5%) | |  |  |  |  |  |  |  |  |
|  |  |  | MVPA: 2.1 min (7.7%) | |  |  |  |  |  |  |  |  |
|  |  |  | Total PA: 7.6 min (28.2%) | |  |  |  |  |  |  |  |  |
|  |  | | | | | | | | | | | |
| 3. | 36 min | | SED: 26.3 min (73.1%) | | Running | | Norwegian (language) | | Indoor  (stairway) | | **Sentences and compound words.** Pupils were given three tasks, while working in groups. 1) Pick up missing words in a sentence to provide meaning to the text, 2) pick up words and write three compound words with the collected words, and 3) each pupil was supposed to write one sentence to a fairytale made up by the group. | |
|  |  |  | LPA: 6.0 min (16.6%) | |  |  |  |  |  |  |  |  |
|  |  |  | MVPA: 3.7 min (9.6%) | |  |  |  |  |  |  |  |  |
|  |  |  | Total PA: 9.7 min (26.9%) | |  |  |  |  |  |  |  |  |
|  |  | |  | |  | |  | |  | |  | |
| 4. | 26 min | | SED: 8.2 min (31.7%) | | Running | | Mathematics | | Outdoor | | **Repetition of plus/minus.** Several tiles with numbers from 0–100 was spread out on a field. By using mental arithmetic and throwing a dice (0-10), groups of pupils was instructed to move from tile to tile according to the previous number plus the number on the dice until they reached 100. Then the same exercise by subtracting the dice number down to 0. | |
|  |  |  | LPA: 8.0 min (30.9%) | |  |  |  |  |  |  |  |  |
|  |  |  | MVPA: 9.7 min (37.4%) | |  |  |  |  |  |  |  |  |
|  |  |  | Total PA: 17.7 min (68.3%) | |  |  |  |  |  |  |  |  |
|  |  | | | | | | | | | | | |
| 5. | 19 min | | SED: 7.7 min (40.6%) | | Running and throwing | | English  (language) | | Outdoor  (ball pit) | | **English glossary.**  Pupils played a ball game, where they threw soft balls at each other. Those that were hit, ran and picked up a cone with an English word. Afterwards they ran to the teacher and told the Norwegian meaning of that word. The teacher approved or disapproved the answer, and the pupil continued in the ball game. | |
|  |  |  | LPA: 6.1 min (32.2%) | |  |  |  |  |  |  |  |  |
|  |  |  | MVPA: 5.2 min (27.2%) | |  |  |  |  |  |  |  |  |
|  |  |  | Total PA: 11.3 min (59.4%) | |  |  |  |  |  |  |  |  |
|  |  | |  | |  | |  | |  | |  | |
| 6. | 26 min | | SED: 21.0 min (80.9%) | | Walking | | Norwegian (language) | | Classroom | | **“From book to movie”.** Pupils worked in pairs with a task with three parts. 1) Pupils walked to a table and collected words related to the topic and wrote down the meaning of that word, 2) pupils walked to the table and collected an explanation of a word, and pupils were supposed to find the correct word, and 3) walk to the table and write down movies that were transformed from a book to a movie. | |
|  |  |  | LPA: 3.9 min (15.0%) | |  |  |  |  |  |  |  |  |
|  |  |  | MVPA: 1.1 min (4.1%) | |  |  |  |  |  |  |  |  |
|  |  |  | Total PA: 5.0 min (19.1%) | |  |  |  |  |  |  |  |  |
| **Additional File 2 cont.: Description of all PAL teaching assessed and observed in the 6^th^ grade.** | | | | | | | | | | | | |
|  | Duration | | PA characteristics | | Primary  movement | | Subject | | Location | | Academic task | |
|  | | | | | | | | | | | | |
| 7. | 26 min | | SED: 19.1 min (73.3%) | | Running and jumping | | Mathematics | | Indoor (stairway) | | **Geometry and figures.** The teacher had placed several exercises in or nearby a large stairway. Groups of pupils were traveling to one exercise and ran back to the bottom of the stairs and solved the task. Afterwards, they continued to the next exercise. | |
|  |  |  | LPA: 4.0 min (15.4%) | |  |  |  |  |  |  |  |  |
|  |  |  | MVPA: 3.0 min (11.4%) | |  |  |  |  |  |  |  |  |
|  |  |  | Total PA: 7.0 min (26.8%) | |  |  |  |  |  |  |  |  |
|  | | | | | | | | | | | | |
| 8. | 20 min | | SED: 10.4 min (51.8%) | | Walking | | English (language) | | Classroom | | **English glossary.** Different English words were put on the walls inside the classroom and hallway. The teacher gave pairs of pupils one word at a time, and the pupils were supposed to find the correct word and choose the correct way of spelling that word from three alternatives. Each spelling alternative from all given words had a letter connected to it, and at the end these letters made up an English word that the pupils were supposed to spell out. | |
|  |  |  | LPA: 6.0 min (29.9%) | |  |  |  |  |  |  |  |  |
|  |  |  | MVPA: 3.7 min (18.3%) | |  |  |  |  |  |  |  |  |
|  |  |  | Total PA: 9.7 min (48.2%) | |  |  |  |  |  |  |  |  |
|  | | | | | | | | | | | | |
| 9. | 28 min | | SED: 22.8 min (81.4%) | | Walking | | Norwegian (language) | | Classroom | | **Sami culture.** Pupils were supposed to color the Sami flag, and to do that correctly they had to walk to the hallway and look at the correct coloring from a picture on the wall. Afterwards, pupils worked in pairs and were supposed to walk up to a table and write down what they associated with Sami culture. | |
|  |  |  | LPA: 4.0 min (14.3%) | |  |  |  |  |  |  |  |  |
|  |  |  | MVPA: 1.2 min (4.3%) | |  |  |  |  |  |  |  |  |
|  |  |  | Total PA: 5.2 min (18.6%) | |  |  |  |  |  |  |  |  |
|  | | | | | | | | | | | | |
| 10. | 26 min | | SED: 21.8 min (83.9%) | | Walking | | Mathematics | | Classroom | | **Fraction calculation.** The teacher had put up different mathematical tasks on the walls. In pairs, pupils were instructed to move around to solve these tasks by discussing possible solutions with each other. Teacher went to each pair to provide supervision. | |
|  |  |  | LPA: 3.2 min (12.3%) | |  |  |  |  |  |  |  |  |
|  |  |  | MVPA: 1.0 min (3.8%) | |  |  |  |  |  |  |  |  |
|  |  |  | Total PA: 4.2 min (16.1%) | |  |  |  |  |  |  |  |  |
|  | | | | | | | | | | | | |
| 11. | 22 min | | SED: 14.1 min (64.1%) | | Running | | English (language) | | Indoor (stairway) | | **English glossary.** English words are put in a cup at the top of a large stairway. Pupils worked in groups and were collecting one word at a time and were supposed to explain each word for each other at the bottom of the stairs without using the actual word. Afterwards, they were supposed to collect the same words, but then put them in sentences to provide meaning to a text. | |
|  |  |  | LPA: 5.6 min (25.5%) | |  |  |  |  |  |  |  |  |
|  |  |  | MVPA: 3.3 min (15.0%) | |  |  |  |  |  |  |  |  |
|  |  |  | Total PA: 8.9 min (40.5%) | |  |  |  |  |  |  |  |  |
|  | | | | | | | | | | | | |
| 12. | 28 min | | SED: 19.0 min (67.7%) | | Walking | | Mathematics | | Indoor  (stairway) | | **Fraction calculation.** Five different math tasks were hidden around and in a large stairway. In groups, pupils were supposed to walk around and find the exercises, read them, and return to the bottom of the stairs and solve it together. After the teacher approved the solution, they walked to find the next exercise. | |
|  |  |  | LPA: 5.9 min (21.0%) | |  |  |  |  |  |  |  |  |
|  |  |  | MVPA: 3.2 min (11.3%) | |  |  |  |  |  |  |  |  |
|  |  |  | Total PA: 9.1 min (32.3%) | |  |  |  |  |  |  |  |  |
|  | | | | | | | | | | | | |
| **Additional File 2 cont.: Description of all PAL teaching assessed and observed in the 6^th^ grade.** | | | | | | | | | | | | |
|  | | Duration | | PA characteristics | | Primary  movement | | Subject | | Location | | Academic task |
|  | | | | | | | | | | | | |
| 13. | | 45 min | | SED: 31.1 min (69.0%) | | Running | | Mathematics | | Outdoor (school-yard) | | **Fraction calculation.** Eleven tasks were hidden under cones, and the pupils were supposed to run up to each cone and collect the task. Back with the group, pupils came up with a solution. The correct answer gave a letter that belonged to a word. After all tasks were solved, the groups were supposed to collect all letters and find put them together to a word. |
|  |  |  |  | LPA: 7.3 min (16.2%) | |  |  |  |  |  |  |  |
|  |  |  |  | MVPA: 6.6 min (14.7%) | |  |  |  |  |  |  |  |
|  |  |  |  | Total PA: 13.9 min (30.9%) | |  |  |  |  |  |  |  |
|  | | | | | | | | | | | | |
| 14. | | 42 min | | SED: 22.7 min (54.0%) | | Running | | Mathematics | | Outdoor (wood) | | **Problem solving.** Ten tasks were placed in a nearby wood and parts of the schoolyard. In pairs, pupils traveled from task to task and solved each task. The tasks included both fraction calculation, basic mathematical exercises and mathematical problem solving. |
|  |  |  |  | LPA: 9.5 min (22.7%) | |  |  |  |  |  |  |  |
|  |  |  |  | MVPA: 9.8 min (23.3%) | |  |  |  |  |  |  |  |
|  |  |  |  | Total PA: 19.3 min (46.0%) | |  |  |  |  |  |  |  |
|  | | | | | | | | | | | | |
| 15. | | 18 min | | SED: 8.3 min (46.1%) | | Running | | English (language) | | Indoor (stairway) | | **English glossary.** The teacher gave the pupils words in Norwegian. The pupils were supposed to find the correct word in English some place in the stairway or in nearby hallways in groups. Each English word had a corresponding code that the pupils had to remember and give to the teacher. |
|  |  |  |  | LPA: 4.6 min (25.5%) | |  |  |  |  |  |  |  |
|  |  |  |  | MVPA: 5.1 min (28.4%) | |  |  |  |  |  |  |  |
|  |  |  |  | Total PA: 9.7 min (53.9%) | |  |  |  |  |  |  |  |
| Values of physical activity are presented as mean and percentage of the PAL session in parentheses. PA, physical activity. SED, sedentary time, LPA, light physical activity. MVPA, moderate-to-vigorous physical activity. | | | | | | | | | | | | |
