# Supplementary material for: Physical activity and content in a variety of physically active learning: an observational case study of real-world practices
Source: Front Sports Act Living. 2025 Jan 3;6:1504704. doi: 10.3389/fspor.2024.1504704 (PMC11739032; doi:10.3389/fspor.2024.1504704)
Supplement: Supplementary file 3 [file Table3.docx]

| **Additional File 3: Description of all PAL teaching assessed and observed in the 8^th^ grade.** | | | | | | |
| --- | --- | --- | --- | --- | --- | --- |
|  | Duration | PA characteristics | Primary  movement | Subject | Location | Academic task |
|  |  | | | | | |
| 1. | 18 min | SED: 10.0 min (55.1%) | Running | English (language) | Outdoor  (school-yard) | **Great Britian.** The teacher gave groups of pupils different questions about Great Britain. The exercise started with a dice throw, where the number indicated a physical exercise (e.g., 5 push-ups, 10 jumping jacks) the group had to perform. One pupil ran across the schoolyard to collect information to solve the questions given by the teacher. This procedure is repeated until all questions were answered. |
|  |  | LPA: 7.5 min (25.1%) |  |  |  |  |
|  |  | MVPA: 3.6 min (19.8%) |  |  |  |  |
|  |  | Total PA: 11.1 min (44.9%) |  |  |  |  |
|  |  |  |  |  |  |  |
| 2. | 27 min | SED: 14.4 min (53.2%) | Running | Mathematics | Outdoor (school-yard) | **Basic mathematical calculations.** Pupils received a map of 15 tasks placed all around the school. In pairs, pupils were supposed to run to one task and solve it and then return to the teacher. When the task was approved, the pupils could continue to the next task. |
|  |  | LPA: 5.2 min (19.6%) |  |  |  |  |
|  |  | MVPA: 7.1 min (26.7%) |  |  |  |  |
|  |  | Total PA: 12.3 min (46.8%) |  |  |  |  |
|  |  | | | | | |
| 3. | 18 min | SED: 11.4 min (63.4%) | Strength exercises | English (language) | Classroom | **Retell and write.** Pupils worked in pairs. One pupil walked out in the hallway and received an English sentence from the teacher. The other pupil was supposed to do a strength exercise until the other pupil returned. The sentence was written down then the pupil from the hallway returned. Afterwards, the pupils switched. |
|  |  | LPA: 3.5 min (19.5%) |  |  |  |  |
|  |  | MVPA: 3.1 min (17.1%) |  |  |  |  |
|  |  | Total PA: 6.6 min (36.6%) |  |  |  |  |
|  |  |  |  |  |  |  |
| 4. | 15 min | SED: 9.7 min (64.6%) | Strength exercises | Mathematics | Classroom | **Topic: Individual work with mathematical tasks.**  All pupils worked at their desk with mathematical tasks. Every 10. min the teacher stopped the work and the pupils followed four short videos (2 – 7 min each) with physical exercises together with the teacher. PA functioned as breaks from academic content. |
|  |  | LPA: 3.1 min (20.5%) |  |  |  |  |
|  |  | MVPA: 2.2 min (14.8%) |  |  |  |  |
|  |  | Total PA: 5.3 min (35.4%) |  |  |  |  |
|  |  | | | | | |
| 5. | 28 min | SED: 11.8 min (42.1%) | Running and throwing | English  (language) | Indoor  (gym) | **Roald Dahl.** Pupils played a ball game in the gym, where they threw soft balls at each other. Those that were hit were given penalty exercise (five squats). Now and then the teacher stopped the game to give the pupils factual sentences about Roald Dahl that they were supposed to retell to another pupil. After that, they returned to the game. PA functioned as breaks from academic content. |
|  |  | LPA: 8.1 min (29.1%) |  |  |  |  |
|  |  | MVPA: 8.1 min (28.9%) |  |  |  |  |
|  |  | Total PA: 16.2 min (58.0%) |  |  |  |  |
|  |  |  |  |  |  |  |
| 6. | 16 min | SED: 8.0 min (49.9%) | Running | English (language) | Indoor  (gym) | **Linking words.** Pupils were playing a game called “The fox and the chicken family”. The “fox” was supposed to catch the pupil placed at the back of a chain of pupils. Pupils switched between being the “fox” and being in the “chicken family”. Now and then the teacher stopped the game, and the pupils ran and wrote down one linking word into a familiar text. This was continued until the text were completed. |
|  |  | LPA: 4.3 min (26.8%) |  |  |  |  |
|  |  | MVPA: 3.7 min (23.3%) |  |  |  |  |
|  |  | Total PA: 8.0 min (50.1%) |  |  |  |  |
| **Additional File 3 cont.: Description of all PAL teaching assessed and observed in the 8^th^ grade.** | | | | | | |
|  | Duration | PA characteristics | Primary  movement | Subject | Location | Academic task |
|  | | | | | | |
| 7. | 15.5 min | SED: 7.8 min (50.7%) | Running  and throwing | English (language) | Indoor (gym) | **“Presentation”.** Pupils played a ball game in the gym, where they threw soft balls at each other. Those that were hit were given penalty exercise (10 jumping jacks). Repeatedly, the teacher stopped the game to give the pupils words related to a good presentation. The pupils were supposed to retell to another pupil and wrote down the word. PA functioned as breaks from academic content. |
|  |  | LPA: 3.4 min (21.8%) |  |  |  |  |
|  |  | MVPA: 4.2 min (27.5%) |  |  |  |  |
|  |  | Total PA: 7.6 min (49.3%) |  |  |  |  |
|  | | | | | | |
| 8. | 31 min | SED: 20.1 min (64.8%) | Walking | Mathematics | Outdoor (school-yard) | **Calculation exercises.** All pupils worked individually with math tasks at their desk. Half of the group were taken outside to play a ball game. This game consisted of keeping a large ball up in the air as long as possible by cooperation. After some time, the group switched with the group inside the classroom. PA functioned as breaks from academic content. |
|  |  | LPA: 6.4 min (21.1%) |  |  |  |  |
|  |  | MVPA: 4.4 min (14.1%) |  |  |  |  |
|  |  | Total PA: 10.8 min (35.2%) |  |  |  |  |
|  | | | | | | |
| 9. | 20 min | SED: 8.4 min (42.0%) | Running and throwing | English  (language) | Indoor  (gym) | **Topic: English writing.**  Pupils played a ball game in the gym, where they threw soft balls at each other. Those that were hit were given penalty exercise (jumping 10 times). Now and then the teacher stopped the game to give the pupils specific English sentences that they were supposed to write down on a paper. After that, they returned to the game. PA functioned as breaks from academic content. |
|  |  | LPA: 5.4 min (26.9%) |  |  |  |  |
|  |  | MVPA: 6.2 min (31.1%) |  |  |  |  |
|  |  | Total PA: 11.6 min (58.0%) |  |  |  |  |
|  | | | | | | |
| 10. | 30 min | SED: 19.5 min (64.8%) | Walking | Mathematics | Outdoor (school-yard) | **Calculation exercises.** All pupils worked individually with math tasks at their desk. Half of the group were taken outside to play a ball game. This game consisted of keeping a large ball up in the air as long as possible by cooperation. After some time, the group switched with the group inside the classroom. PA functioned as breaks from academic content. |
|  |  | LPA: 7.0 min (23.3%) |  |  |  |  |
|  |  | MVPA: 3.5 min (11.8%) |  |  |  |  |
|  |  | Total PA: 10.5 min (35.1%) |  |  |  |  |
| Values of physical activity are presented as mean and percentage of the PAL session in parentheses. PA, physical activity. SED, sedentary time, LPA, light physical activity. MVPA, moderate-to-vigorous physical activity. | | | | | | |
